# Dedicated Cone-Beam Breast CT: Reproducibility of Volumetric Glandular Fraction with Advanced Image Reconstruction Methods

**DOI:** 10.3390/tomography9060160

**Published:** 2023-11-02

**Authors:** Srinivasan Vedantham, Hsin Wu Tseng, Zhiyang Fu, Hsiao-Hui Sherry Chow

**Affiliations:** 1 Department of Medical Imaging, University of Arizona, Tucson, AZ 85724, USA; tseng45@radiology.arizona.edu (H.W.T.); zyfu@arizona.edu (Z.F.); 2 Department of Biomedical Engineering, University of Arizona, Tucson, AZ 85724, USA; 3 Department of Medicine, University of Arizona, Tucson, AZ 85724, USA; schow@arizona.edu

**Keywords:** breast cancer, screening, breast CT, deep learning, artificial intelligence, breast density

## Abstract

Dedicated cone-beam breast computed tomography (CBBCT) is an emerging modality and provides fully three-dimensional (3D) images of the uncompressed breast at an isotropic voxel resolution. In an effort to translate this modality to breast cancer screening, advanced image reconstruction methods are being pursued. Since radiographic breast density is an established risk factor for breast cancer and CBBCT provides volumetric data, this study investigates the reproducibility of the volumetric glandular fraction (VGF), defined as the proportion of fibroglandular tissue volume relative to the total breast volume excluding the skin. Four image reconstruction methods were investigated: the analytical Feldkamp–Davis–Kress (FDK), a compressed sensing-based fast, regularized, iterative statistical technique (FRIST), a fully supervised deep learning approach using a multi-scale residual dense network (MS-RDN), and a self-supervised approach based on Noise-to-Noise (N2N) learning. Projection datasets from 106 women who participated in a prior clinical trial were reconstructed using each of these algorithms at a fixed isotropic voxel size of (0.273 mm^3^). Each reconstructed breast volume was segmented into skin, adipose, and fibroglandular tissues, and the VGF was computed. The VGF did not differ among the four reconstruction methods (*p* = 0.167), and none of the three advanced image reconstruction algorithms differed from the standard FDK reconstruction (*p* > 0.862). Advanced reconstruction algorithms developed for low-dose CBBCT reproduce the VGF to provide quantitative breast density, which can be used for risk estimation.

## 1. Introduction

Mammography is a well-established standard for breast cancer screening. Screening mammography followed by adjuvant therapy has demonstrated mortality reduction [1,2]. The transition to digital mammography from screen-film mammography provided additional benefits including a wider dynamic range, the ease of post-processing and computer-aided detection techniques. Overall, the diagnostic accuracy of screen-film and digital mammography is similar for breast cancer screening, with digital mammography being more accurate in premenopausal or perimenopausal women, women under the age of 50 years, and women with radiographically dense breasts [3]. However, mammography suffers from two major limitations. First, the sensitivity of mammography is substantially reduced for women with dense breasts [4]. Hence, adjunctive screening with ultrasound is recommended for women with dense breasts. While handheld ultrasound is widely used, automated breast ultrasound [5] and ultrasound tomography [6] (UST) systems are also available. Adjunctive screening with ultrasound has demonstrated an improved cancer detection rate [5,7]. The second limitation of mammography is that it suffers from tissue superposition and results in missed cancers or unnecessary recalls for additional imaging. To overcome this limitation, digital breast tomosynthesis (DBT) [8], which produces quasi-3D images, was introduced. DBT has demonstrated a reduced recall rate and increased cancer detection rate [9]. In addition to the above modalities, dynamic contrast-enhanced MRI (DCE-MRI) is also recommended for women with a high life-time risk for breast cancer due to its high sensitivity for detecting breast lesions [10].

Dedicated breast computed tomography (BCT) is an emerging imaging modality [11,12,13]. The idea of performing computed tomography of the breast was envisioned in the mid-1970s and clinical prototype systems were built. In a study of 1625 patients, BCT detected 94% of the 78 cancers, whereas mammography detected 77% of the cancers [14]. However, due to the technology available at that time, there were several major limitations that impeded its translation to the clinic. First, it was a single slice scanner using a high-pressure xenon gas detector with a limited dynamic range. This necessitated the intravenous administration of iodinated contrast media. Also, a water bath surrounding the breast was needed to avoid saturating the detector. Second, the in-plane voxel size was 1.56 × 1.56 mm and the slice thickness was 1 cm and hence suffered from poor spatial resolution. Third, the radiation dose from the imaging exam was approximately 4–5 times that of mammography. Since the hardware and the technology for CT imaging substantially improved in the 1980s and 1990s, the feasibility of performing BCT was revisited with specimens in the mid-1990s [15]. The study showed improved confidence to detect soft tissue lesions in dense breast tissue with CT compared with surgical specimen radiography but was inferior for detecting microcalcification clusters. The study did not provide dose estimates or compare with mammography. A major impetus to the field of BCT was the article by Boone et al. [16] that showed that the mean glandular dose (MGD) comparable to mammography was achievable with BCT.

Modern BCT systems provide fully 3D images at an isotropic voxel resolution. They image the uncompressed breast and hence alleviate the discomfort associated with breast compression in mammography and DBT. The fully 3D images eliminate the tissue superposition observed with mammography, which contributes to false positives (unnecessary recall) and false negatives (missed cancers). The fully 3D images from BCT also eliminate “out-of-slice” artifacts associated with limited-angle acquisition in DBT [17]. Broadly, based on the image acquisition method, BCT can be classified as cone-beam breast CT [18,19,20], helical breast CT [21], or slot-scan breast CT [22]. A majority of the clinical and prototype breast CT systems are based on the cone-beam CT approach. In the United States, cone-beam breast CT is FDA-approved for diagnostic imaging [23]. In the diagnostic setting, a multi-reader multi-case study in which each of the 18 radiologists interpreted both cone-beam breast CT (CBBCT) and diagnostic mammograms from 235 cases, of which approximately half of the cases had calcified findings, demonstrated improved sensitivity with CBBCT and a similar specificity and area under the receiver operating characteristic (ROC) curve compared with mammography-based diagnostic workup [24].

In an effort to translate BCT to breast cancer screening, multiple research teams and industrial partners are working on various hardware approaches including the use of high-resolution, low-noise, complementary metal oxide semiconductor (CMOS) detectors [25] either with full-scan [25,26] or short-scan acquisition [27,28], or photon-counting detectors with helical acquisition [29]. The primary objective of these research efforts is to reduce the radiation dose so that it is appropriate for screening exams while improving the visualization of microcalcifications.

In breast X-ray imaging (mammography, DBT, BCT), the radiation dose to the breast is uniformly reported using the metric, Mean Glandular Dose (MGD). This metric apportions the radiation dose to the fibroglandular tissue, which is at risk for developing breast cancer [30]. The Mammography Quality Standards Act (MQSA) limits the MGD to 3 mGy for the cranial-caudal (CC) view of a phantom that represents an average-sized breast [31]. A standard screening exam comprises two views: the CC and mediolateral oblique (MLO) view. The MGD from a standard two-view screening mammogram is approximately 4.15–4.98 mGy [32]. There is a marginal difference in the MGD when screened with DBT alone, and an approximate doubling of the MGD when screened with both DBT and mammography [8,33]. For diagnostic imaging using mammography and DBT, the MGD is dependent on the number of views and on average is approximately 12–16 mGy [34,35]. In the diagnostic setting, the MGD with an early generation CBBCT (13.9 ± 4.6 mGy) was similar to, and within the range of, mammography-based diagnostic workup (12.4 ± 6.3 mGy) [34]. Subsequent improvements have reduced the radiation dose to approximately 7.2 ± 2.6 mGy [19,36]. Since BCT replaces these two standard views with a single scan, an MGD in the range of 3 to 6 mGy for breast cancer screening with BCT is generally considered to be reasonable. MQSA requires the visualization of 320 μm calcification clusters. The current generation of BCT systems achieves a sufficient spatial resolution to enable the detection of these calcification clusters [25,29,37].

Radiographic breast density is an established risk factor for breast cancer [38]. Studies have reported over a four-fold increased risk of breast cancer for women with the most dense breasts compared with the least dense breasts [38,39,40]. Clinically, breast density is reported using a four-point categorical scale based on radiologists’ impression of the spatial distribution (area) of fibroglandular tissue and it suffers from inter-reader variability. Several commercial and research tools [41,42,43,44,45] are available for computing volumetric breast density or for estimating the risk for breast cancer from mammography and digital breast tomosynthesis images. Since BCT provides volumetric data, it is readily suitable for quantifying the volumetric fibroglandular fraction (VGF) [46], which is also referred to as the volumetric breast density [47]. The VGF is defined as the ratio of the fibroglandular tissue volume to the total breast volume after excluding the skin. All clinical studies with CBBCT reported to date use the analytical Feldkamp–Davis–Kress (FDK) algorithm [48] for image reconstruction. In an effort to reduce the radiation dose while maintaining the image quality, advanced image reconstruction algorithms are being investigated by several research groups [49,50,51,52]. This study investigates if the VGF is reproducible across these advanced image reconstruction algorithms.

## 2. Materials and Methods

### 2.1. Human Subjects

This retrospective study utilized de-identified projection datasets from 106 research participants, all women, who participated in a prior institutional review board (IRB)-approved, health insurance portability and accountability act (HIPAA)-compliant clinical trial (clinicaltrials.gov identifier: NCT01090687). At the time of the clinical trial, all subjects provided written informed consent. Our IRB determined that the use of this de-identified dataset which has been shared with multiple institutions does not constitute human subjects research (UA protocol # 1903470973). These datasets were acquired on a pre-FDA approval prototype (Koning Corp., Norcross, GA, USA). The system uses a CsI:Tl scintillator-coupled amorphous silicon flat-panel detector (PaxScan^®^ 4030CB, Varex Imaging, Salt Lake City, UT, USA) and a mammography-format X-ray tube (RAD71-SP, Varex Imaging, Salt Lake City, UT, USA). Projection images were acquired using a 0.3 mm (nominal) focal spot size at 49 KV, and the measured half-value layer thickness of the X-ray beam was 1.4 mm of Al. The X-ray source operated in pulsed mode with an 8 ms pulse width. The tube current (mA) varied with breast size and composition and was determined from two orthogonal scout views. The MGD varies with tube current in a linear manner. The detector had a native detector element size of 0.194 mm and was operated in a 2 × 2 binned mode to provide 0.388 mm-sized detector elements and the projection images were acquired at 30 frames (or views) per second. A total of 300 projections were acquired during the full-scan (360-degree rotation of the X-ray source and detector) and the total scan time was 10 s. Additional details of this CBBCT system can be found in prior publications [18,46].

### 2.2. Image Reconstruction

The projection datasets were reconstructed to a fixed isotropic voxel pitch of 0.273 × 0.273 × 0.273 mm^3^ using four image reconstruction algorithms. These include the well-known analytical FDK algorithm [48], which is the standard algorithm used for clinical interpretation; a compressed sensing-based fast, iterative, total-variation regularized, statistical technical (FRIST) algorithm [26,27,50]; an artificial intelligence-based fully supervised deep learning algorithm that used a multi-scale residual dense network (MS-RDN) architecture [52]; and an artificial intelligence-based self-supervised deep learning algorithm that used the Noise-to-Noise (N2N) paradigm [53] and the residual dense network structure employed in MS-RDN.

The FRIST algorithm implements the adaptive steepest descent-projection onto convex sets (ASD-POCS) that was originally described by Sidky and Pan [54] and is based on rigorous math, with a focus on practical improvements to enable clinical translation. Our investigations [50] showed that, using the original algorithm [54], we needed to reconstruct to quarter voxel-size (in-plane) followed by down-sampling to reduce aliasing/interference-like artifacts [55]. This was impractical even with GPU acceleration due to the high-resolution BCT data [50]. Hence, we conducted a systematic evaluation focused on practicality. In addition to GPU acceleration, we made FRIST practically feasible by initializing with an FDK image and replacing the algebraic reconstruction technique (ART) with the ordered subsets—simultaneous algebraic reconstruction technique (OS-SART).

The fully supervised MS-RDN architecture described in our prior work [52] has been shown to generate images with sharper fine structures than residual encoder-decoder convolutional neural network (RED-CNN) [56]. The combination of residual connections and densely connected structures used in residual dense blocks has been shown to improve network parameter efficiency and reconstruction accuracy. Importantly, this algorithm can be tuned based on a physics-based measure of slice-sensitivity profile as multiple adjacent slices are provided as input-label pairs. The detector used for data acquisition employed a CsI:Tl scintillator to convert X-rays to light photons, and these light photons spread to adjacent slices; hence, there are correlations between adjacent slices. This network exploits these correlations through multi-slice training. Evaluation of this network architecture and physics-based tuning indicated that including 5 adjacent slices improved performance. The fully supervised MS-RDN was trained patch-wise on a large dataset of 8346 input-label pairs of image slices. The inputs to the network are multi-slice patches randomly extracted from the sparse-view (100 views) FDK reconstructions, and the labels of the network are the corresponding patches extracted from the full-scan (300 views) FDK reconstructions.

The self-supervised deep learning network uses the Noise-to-Noise (N2N) paradigm [53,57]. N2N is an image-denoising network requiring pairs of independent noisy images of the same object rather than the noisy (input-label) pairs. To further accommodate N2N learning for image inverse problems, we distribute acquired (noisy) data into disjoint sets and reconstruct those using linear analytical methods to obtain independent noisy image pairs. Specifically, we split the projection data (300 projections) equally into odd and even projection views (150 projections each) and reconstructed each of these projection datasets (150 views each) using the FDK algorithm. We adopted the MS-RDN architecture for the self-supervised learning and tuned the network size in terms of the number of layers and the number of residual dense blocks. The network was trained in a pseudo-3D manner: 2D patches of 128 × 128 were randomly extracted along the three orthogonal directions.

### 2.3. VGF Computation

**Figure 1:**
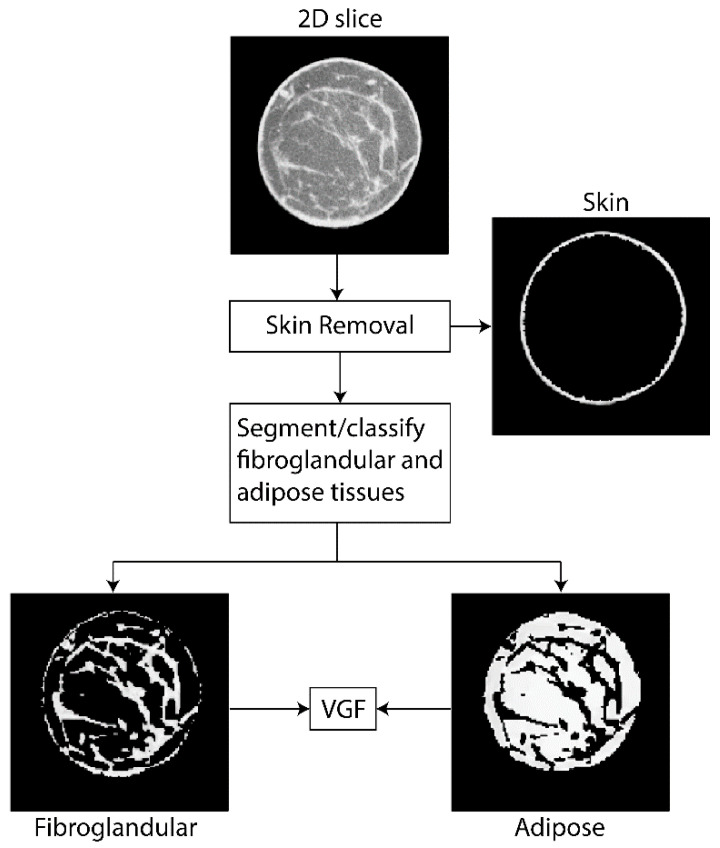
Flowchart of the steps used to compute the volumetric glandular fraction (VGF). From each 2D slice, the skin line is identified and removed. The remaining breast volume is segmented into adipose and fibroglandular tissues, from which the VGF is computed.

From each reconstructed breast volume, the skin was first segmented and removed using the method described in our prior work [58]. The breast volume excluding the skin served as the input for the kernel-based fuzzy C-Means (KFCM) algorithm used for segmentation [46], and each voxel was then classified as adipose or fibroglandular tissue. KFCM is an automated and unsupervised segmentation method. The KFCM method is less affected by outliers and incomplete data. The KFCM was implemented with voxel intensity as the image feature and with a Gaussian kernel. This method was chosen as our prior work [46] using a 3D-printed phantom showed that the method was accurate in quantifying fibroglandular volume and hence the VGF to within ±1.9%. Figure 1 shows an example, where the 2D cross-sectional slice is segmented and classified as skin, fibroglandular, and adipose tissues. Following segmentation and classification, the VGF was computed as the ratio of the fibroglandular tissue volume ($V_{g}$) to the total breast volume ($V_{g}+V_{a}$), where $V_{a}$ represents the volume of adipose tissue in the breast.

### 2.4. Statistical Analysis

The VGF from each reconstruction was tested for normal distribution (Shapiro–Wilk’s test), and appropriate summary statistics were computed. Depending upon whether the VGF distribution satisfied the normality assumption, either repeated measures ANOVA or Friedman’s test was used to test if the VGF differed between the four reconstruction methods. Appropriate multiple comparisons tests were performed to determine if each of the advanced image reconstruction methods differed from the standard FDK reconstruction. Effects associated with *p* < 0.05 were considered statistically significant. All analyses were performed using GraphPad Prism (ver. 10.0.2, GraphPad Software, LLC, Boston, MA, USA).

## 3. Results

Table 1 summarizes the breast dimensions and the BI-RADS breast density assigned from mammography. The distribution included subjects from each of the four BI-RADS breast density categories. For two subjects, the BI-RADS breast density category could not be retrieved. The MGD to each breast was determined by factoring in its dimensions and using the previously described methodology [34]. For the cohort of 106 breasts studied, the mean ± standard deviation of the MGD from the diagnostic, non-contrast CBBCT was 14.4 ± 4.9 mGy and the median (inter-quartile range, IQR) was 13.3 (10.3–17.9) mGy.

**Figure 2:**
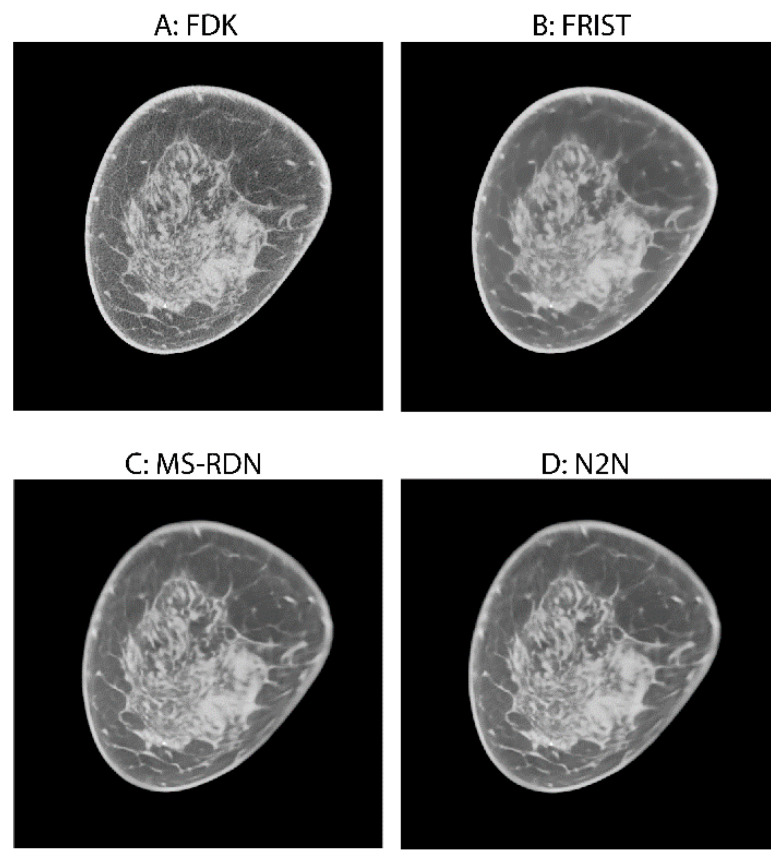
An example showing the cross-sectional images from all four image reconstruction algorithms. (**A**) Analytical FDK reconstruction, which is the reference standard; (**B**) compressed sensing-based FRIST; (**C**) fully supervised deep learning-based MS-RDN; (**D**) self-supervised deep learning-based N2N.

**Figure 3:**
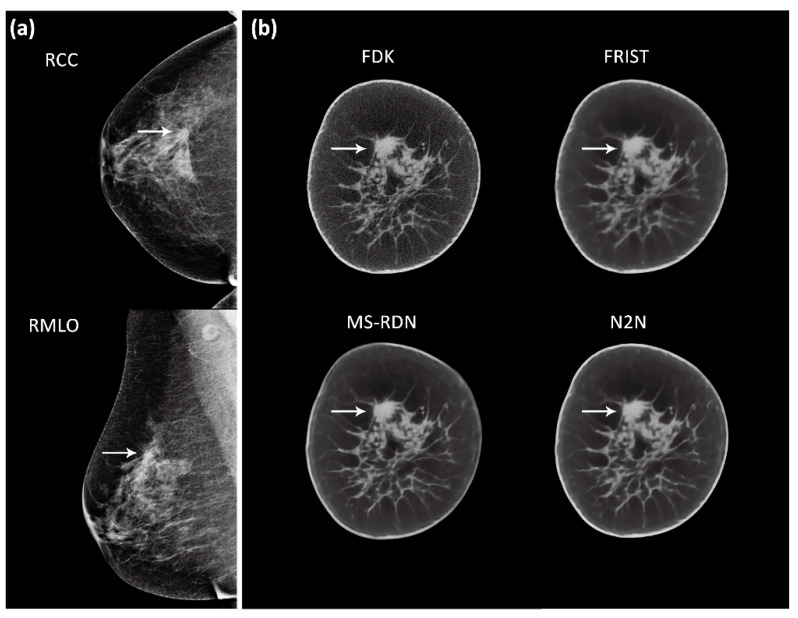
An example showing a soft tissue lesion (arrows) in the right breast (**a**) digital mammography and (**b**) BCT. Five-slice averages from all four BCT image reconstruction algorithms are shown with display window of [0.21, 0.33] cm^−1^ (in units of linear attenuation coefficient). Subsequent to imaging, the lesion was biopsied and was pathology-verified as invasive ductal carcinoma.

**Figure 4:**
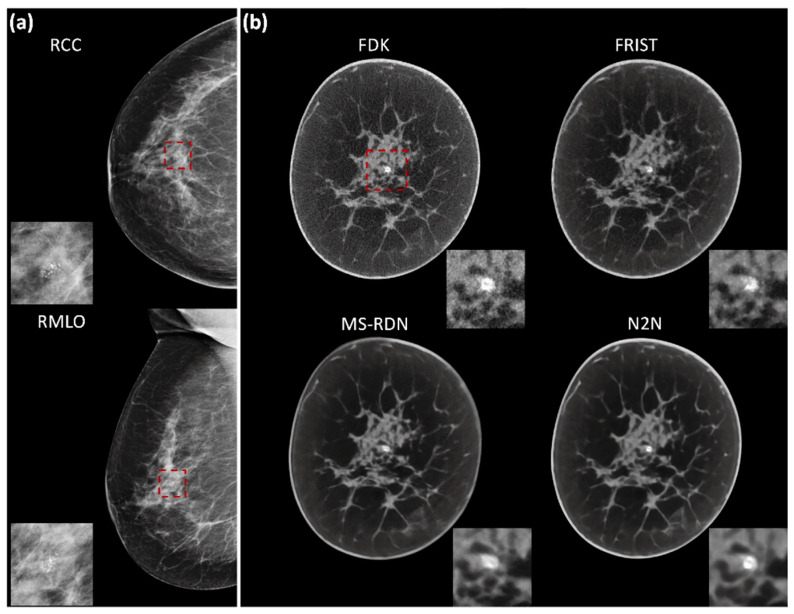
An example showing a microcalcification cluster in the right breast (**a**) digital mammography and (**b**) BCT. Five-slice maximum intensity projections (MIP) from all four BCT image reconstruction algorithms are shown with display window of [0.21, 0.33] cm^−1^. In each panel, the close-up view of the microcalcification cluster (red dashed box) is shown as an insert. Subsequent to imaging, the lesion was biopsied and was pathology-verified as ductal carcinoma in situ (DCIS).

Figure 2 shows an example case with image reconstructions using the four algorithms. In order to demonstrate the visual appearance of lesions in mammograms and BCT reconstructions, example cases are shown in Figure 3 and Figure 4, corresponding to a soft tissue lesion and a microcalcification cluster, respectively. The soft tissue lesion shown in Figure 3 was subsequently biopsied and was pathology-verified to be an invasive ductal carcinoma (IDC). The microcalcification cluster shown in Figure 4 was subsequently biopsied and was pathology-verified to be ductal carcinoma in situ (DCIS).

**Figure 5:**
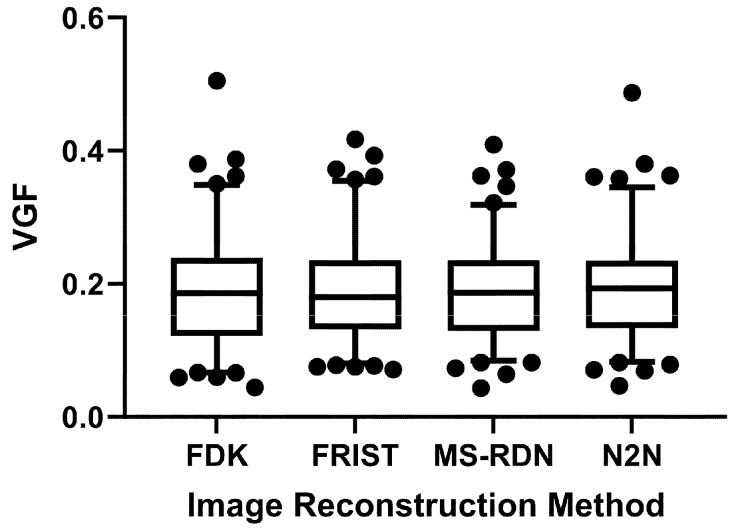
Box plot of the distribution of VGF from all four image reconstruction algorithms. The box ends represent the inter-quartile range and the whiskers the 5–95% of the distribution.

The distribution of VGF from each of the four image reconstructions did not satisfy the normality assumption (*p* < 0.021, Shapiro–Wilk’s test). Hence, the median, IQR, and range of the VGF are summarized in Table 2. For the standard FDK reconstruction, the median, IQR, and range of the VGF were 0.186, (0.122, 0.239), and [0.04–0.505], respectively. For the matched datasets, the VGF did not differ significantly among the four reconstructions (*p* = 0.167, Friedman’s test). Also, the VGF from the advanced image reconstruction algorithms did not differ from the standard FDK reconstruction (*p* > 0.862, Dunn’s multiple comparisons test). The *p*-values for the comparison between the advanced image reconstruction methods and the standard FDK reconstruction are included in Table 2. Figure 5 shows the box plot of the VGF from the four reconstruction methods. The box ends represent the IQR, the central line within the box represents the median, and the whiskers represent the 5–95% of the VGF distribution.

## 4. Discussion

The primary objective of this study was to evaluate if the advanced image reconstruction algorithms that have been developed for low-dose CBBCT affect the quantitative estimate of breast density, the volumetric glandular fraction (VGF). The study included both compressed sensing and deep learning-based image reconstruction algorithms. The two selected deep learning-based image reconstruction algorithms represent a fully supervised learning framework (MS-RDN) and a self-supervised learning framework (N2N). A major advantage of our approach is that the same set of projection data is reconstructed to the same voxel pitch, hence enabling the evaluation of the algorithms at matched conditions. Currently, all CBBCT systems utilize FDK reconstruction and the reconstructed volumetric image data can be used to quantify the VGF. The VGF, either alone or in combination with known clinical and genetic risk factors, can be used to develop models for estimating breast cancer risk estimation. The VGF can also provide a quantitative metric to determine the eligibility for adjunctive screening of women with dense breasts. The results from this study showing that the VGF estimates did not differ among the algorithms and that the VGF from each of the advanced image reconstruction algorithms did not differ from the standard FDK reconstruction is important, as risk estimates developed using VGF from FDK reconstructions can be readily applied even when the images are reconstructed using advanced algorithms.

Several deep learning-based reconstruction methods have received regulatory approval for use in conventional multi-detector CT (MDCT) and these are discussed in a recent topical review [59]. Since these commercially available reconstruction methods are proprietary, details of their implementation have not been disclosed. Broadly, these reconstruction methods can be classified based on type (direct or indirect), learning method (supervised, semi-supervised, self-supervised, and unsupervised), and function (reconstruction, restoration, or denoising). In direct type, the deep learning method performs complete image reconstruction by inverting the system matrix and generating the reconstructed images from projections or sinograms. In the indirect type, the inversion of the system matrix is achieved using established methods such as FDK or iterative reconstruction, and the deep learning method utilizes projection/sinogram data, or reconstructed image data, or both (dual domain) to restore or denoise the image. Both the MS-RDN and the N2N studied correspond to the indirect type and utilize only the reconstructed image domain data. While the MS-RDN performs image restoration, the N2N performs denoising alone. MS-RDN is a fully supervised learning method, whereas N2N uses self-supervised learning. Among the current US FDA-approved deep learning algorithms used in MDCT [59], the MS-RDN has commonalities with AiCE (Canon Medical Systems USA, Inc., Tustin, CA, USA) in that it is fully supervised and is of indirect type using image domain data. The N2N algorithm is functionally similar to ClariCT.AI (ClariPi, Seoul, South Korea) and PixelShine (AlgoMedica Inc., Sunnyvale, CA, USA) in that it denoises the reconstructed image and differs in that it uses self-supervised learning as opposed to fully supervised learning in the commercially available deep learning algorithms.

The major advantage of these advanced image reconstruction algorithms is the ability to denoise the image without degrading its quality. This would allow for the reduction of the radiation dose so that BCT would be suitable for screening. For linear reconstruction algorithms, the achievable dose reduction can be computed from the image variance. However, for non-linear reconstruction algorithms such as the FRIST, MS-RDN, and N2N studied here, the achievable dose reduction cannot be directly estimated from the image variance. Hence, the dose reductions achievable with each of these algorithms are discussed below.

In this study, the compressed sensing-based FRIST algorithm utilized all 300 projections for image reconstruction. Hence, the MGD is 14.4 ± 4.9 mGy. A prior study [50] using the FRIST algorithm showed that the method significantly reduced image noise while maintaining quantitative accuracy and improved the signal-to-noise ratio by 200%. Importantly, no degradation of spatial resolution was observed. In a follow-up study [26] using a high-resolution detector in offset geometry, which corresponds to the latest generation of CBBCT, it was shown that the FRIST reduced the image variance by approximately 59% and improved the signal-to-noise ratio by approximately 38% compared with the FDK reconstruction. The offset-detector geometry results in radiation dose reduction that depends on breast size, with an approximate 6–10% reduction in the MGD for large breasts [60]. Another study demonstrated the feasibility of short-scan, sparse-view breast CT, which can reduce scan times and, consequently, the likelihood of motion artifacts and reduce the radiation dose without degrading the image quality [27]. The study showed that the MGD could be potentially reduced by approximately 45% using short-scan, sparse-view FRIST reconstruction while preserving similar imaging quality to the full-scan FDK reconstruction. While the reconstruction time depends on the computing hardware, it was demonstrated that a single iteration of the FRIST algorithm was comparable to the FDK reconstruction. High-quality reconstructed images are obtained after approximately 80–100 iterations of the FRIST algorithm. It takes approximately 20–25 min to reconstruct an average-sized breast with approximately 450 slices at a slice thickness of 0.273 mm. Substantial acceleration of the image reconstruction can be achieved with hardware improvements and by further parallelizing the reconstruction algorithm.

In this study, the fully supervised learning-based MS-RDN utilized all 300 projections for image reconstruction. Hence, the MGD is 14.4 ± 4.9 mGy. MS-RDN allows for a fast reconstruction speed, which could be useful for image-guided biopsies. Importantly, the multi-slice approach used with MS-RDN leverages correlations between adjacent slices and provides improved image quality. In a prior work, it was demonstrated that the MS-RDN utilizing five adjacent slices outperformed the FRIST in terms of quantitative image quality metrics [52]. The MS-RDN also outperformed the fully supervised RED-CNN. The study showed the potential to reduce the MGD by approximately 66%. Thus, based on the average MGD of 14.4 mGy in this study, an average MGD of 4.8 mGy is achievable and is within the range reported for standard two-view screening mammography [32]. However, the fully supervised learning framework could be susceptible to artifacts when it encounters out-of-distribution data rather than that used for training. Also, the MS-RDN was trained with a pixel-wise loss function which can lead to the over-smoothing of structures in the image. Evaluation of alternate loss functions, such as perceptual loss and texture loss, is the subject of ongoing research.

In this study, the self-supervised learning-based N2N utilized all 300 projections for image reconstruction. Hence, the MGD is 14.4 ± 4.9 mGy. Unlike MS-RDN, N2N is less susceptible to out-of-distribution data. Its reconstruction speed should be even faster than MS-RDN due to the smaller network size. It is relevant to note that the reconstruction speed is less important for screening studies, as these exams are batch-read at most institutions. However, the extra data undersampling/splitting associated with the training of N2N may limit the reconstruction performance at higher undersampling rates as the residual streaking artifacts start to weigh in. Thus, each of the advanced image reconstruction algorithms has its advantages and challenges, and the choice of the reconstruction method could depend on the intended clinical application.

This study only investigated if the VGF differed between the image reconstruction methods. Development of a statistical risk model based on the quantitative VGF would require a much larger sample size and is planned for the future. While this study shows that the VGF from all four reconstruction methods is not different, the selection of the reconstruction method for clinical interpretation will be based on the ability to accurately detect and diagnose breast lesions. This is the subject of ongoing research.

## 5. Conclusions

High-resolution, low-dose dedicated CBBCT is being actively investigated for the potential translation to breast cancer screening. Toward this goal, advanced image reconstruction algorithms are being investigated. Our study shows that the compressed sensing-based iterative FRIST reconstruction, fully supervised learning-based MS-RDN, and self-supervised learning-based N2N methods developed for low-dose CBBCT reproduce the VGF estimated from the standard FDK reconstruction. The VGF provides quantitative estimates of breast density and can be used for modeling the risk for breast cancer. Since all of the CBBCT image data available to date are from FDK reconstructions, a risk model developed using the VGF computed from these reconstructions can also be used with the VGF computed from the investigated advanced reconstruction methods.

## Figures and Tables

**Table 1:** Summary of breast dimensions and BI-RADS breast density assigned from mammography (*n* = 106). For two subjects, the BI-RADS breast density category could not be retrieved.

Diameter of breast at chest-wall	13.1 ± 2.3 cm
Chest-wall to nipple length	9.7 ± 2.8 cm
BI-RADS breast density categories from mammography	
A: Almost entirely fatty	7/104 (7%)
B: Scattered areas of fibroglandular density	39/104 (38%)
C: Heterogeneously dense	35/104 (34%)
D: Extremely dense	23/104 (22%)

**Table 2:** Volumetric glandular fraction (VGF) from four image reconstructions (*n* = 106; IQR—Inter-quartile range; *p*-values are from Dunn’s multiple comparisons test with respect to the standard FDK reconstruction).

Reconstruction Method	Median (IQR) [Range]	*p*-Value
FDK	0.186 (0.122, 0.239) [0.04–0.505]	NA
FRIST	0.18 (0.131, 0.235) [0.07–0.417]	0.936
MS-RDN	0.187 (0.129, 0.235) [0.043–0.409]	0.862
N2N	0.193 (0.133, 0.235) [0.047–0.487]	>0.999

## Data Availability

No new data were created or analyzed in this study. Data sharing is not applicable to this article.
